# Round the Hospitals

**Published:** 1917-01-13

**Authors:** 


### ROUND THE HOSPITALS.

By command of H.R.H. Princess Christian, the President, a special general meeting of the Royal British Nurses' Association will be held in the Lecture Hall of the Royal Society of Medicine, 1 Wimpole Street, Cavendish Square, W., on Thursday, January 18, at 2.30 P.M. The business will be to consider the proposed amalgamation of the Association with the College of Nursing, Limited, in accordance with an agreement, dated 21st ultimo, the confirmation of which will be submitted to the meeting for approval. In the ordinary course, as the British nursing world expects and desires, this amalgamation would, and we believe will, be carried by an overwhelming majority, because British nurses are united to-day in working with a will to secure for themselves and their profession a Royal College of Nursing, State Registration, and the Statutory Recognition of their profession. It is rumoured that the little party of "Divide and Delay," led by the lady, will attempt to raise the voice of discord once more at the Royal British Nurses' Association meeting. If this proves true, the malcontents should meet their deserts at the hands of the speakers and audience. Is there one fully-trained nurse who can be persuaded, by tarradiddles and make-believe, to exhibit herself as an opponent to the immediate creation of a Royal British College of Nursing, which the proposed amalgamation, if approved, will make an accomplished fact on the 18th instant?

---

It is very much to be regretted that Miss Haughton, the Matron of Guy's Hospital, should be prevented by her serious illness from taking part in the organisation of Irish nursing at the present time. Miss Haughton was formerly Lady Superintendent of Sir Patrick Dun's Hospital, Dublin, has an intimate knowledge of Irish nursing, and is deservedly held in the highest esteem there. We are glad to be able to record that Miss Haughton is making steady progress. The sympathy of the whole nursing world is with her. Everybody prays and hopes for her complete recovery. Irish nursing affairs are becoming active at the moment, and arrangements have now been made for Miss Cox-Davies and Miss Rundle to visit Ireland at the end of the month as representatives of the College of Nursing. The first meeting which Miss Cox-Davies will address will be held in Dublin on Saturday afternoon, the 27th inst, at the Royal College of Physicians, the President, Dr. J. O'Carroll, being in the chair. There will be a second meeting, at Belfast, on Monday evening, January 29, and it is anticipated that both will be largely attended and full of interest. It is possible that a meeting may be held at Cork, provided that the matrons and nurses there wish it, and speedily make their wishes known to the College authorities.

---

We hope the *Irish Weekly Times* and other papers will give Irish nurses an opportunity of understanding the present position of nursing matters in relation to the College of British Nursing, by republishing our article on "The Nation and its Nurses," which appeared in THE HOSPITAL of the 6th instant, page 285. They should further bear in mind that, the sowers of disunion in the nursing body have now frankly shown their true character and aims, by giving themselves the name of Professional Registrationsts. Those who are opposed to these agitators are disinterested, whole-hearted supporters of nursing, and have no axes whatever to grind, their sole aim and object being to secure the Highest Standard of Training, the Adequate Protection of the Nurse, and the granting of Protection with an Adequate Status to her profession. They are the upholders of the Principles enforced and taught by Florence Nightingale, which certainly did not include the employment of the services of Professional Registrationists and agitators, with political aims, in association with British nursing. Neither Parliament, the Public, nor the Working Nurses, certainly in this country and we hope in Ireland, are likely to imperil their birthright and position in the nursing world, by entrusting their interests to Professional Registrationists with axes to grind, whose personal ambitions and thirst for supreme power over the nurses and the nursing profession, are so generally recognised in this country, as to make them impotent for serious mischief, either now or in the future.

**ROUND THE HOSPITALS—** *(continued).*

Mrs. Dawson Scott, of the Women's Defence Relief Corps, asks us to point out to students, nurses, and hospital workers who may wish to spend a portion of their holidays working with farmers on the land, that any one of them willing to do similar work this year should write, enclosing a stamped envelope and intimating their willingness to join a party for farm work, to Miss Myers, Organiser, 10 Abbey Road, London, N.W. Mrs. Dawson Scott points out that more women will be needed on the land this year, and that the Corps likes to organise its parties in advance, so as to have everything well arranged and comfortable for the workers. Mrs. Dawson Scott makes it a rule to send a visitor to inspect the accommodation, and the Corps insists on a living wage being paid to each worker. We may add that at the request of the President of the Board of Agriculture, Mrs. Dawson Scott and her Corps are working in conjunction with the Women's National Land Service Corps.

Statements in the daily press have raised the question of a shortage of nurses. Is there any such shortage in reality in this country at the present time? We have been making inquiries, and have reason to declare that where an institution with vacancies is ably administered and of good repute there is no shortage of sisters and staff nurses to fill such vacancies as may occur from time to time. Where vacancies have occurred, one matron has written to say that an advertisement in *The Nursing Mirror* produced twenty applications for a single vacancy in twenty-four hours. The Colonial Nursing Association finds no falling off in the supply of nurses at the present time, though war conditions might reasonably be supposed to cause one. It is said that the supply of trained nurses for civil work is likely to increase rather than to diminish, from the circumstance that experience in the nursing of wounded warriors proves its arduous character, and that after two years of it many nurses find it desirable to seek a change of work by making their way back to the wards of a civil hospital. We are glad to be assured that at present, at any rate, this has not affected the supply of nurses for Army purposes. On the other hand, some Poor-Law matrons have found the supply of probationers much less than formerly. This, we think, is a matter which will adjust itself quite readily in due course. In the case of nurses required for district work, especially in the country, there is in several directions a great shortage at the present time. This shortage is just and right, seeing that district nurses on the whole are frequently gravely underpaid, though salaries in the country might really be higher than those in the cities and towns, as a compensation for many disadvantages attaching to this special work. No qualified nurse who devotes herself to district work should receive less than £100 a year. These facts lead to the conclusion that there is no shortage of nurses among the hospitals and kindred institutions, and that any shortage there is in district and other nursing work may be due to inadequate remuneration or some special cause, which an able administrator would remove with a stroke of the pen.

